# Using the Electroretinogram to Understand How Intraocular Pressure Elevation Affects the Rat Retina

**DOI:** 10.1155/2013/262467

**Published:** 2013-01-29

**Authors:** Bang V. Bui, Zheng He, Algis J. Vingrys, Christine T. O. Nguyen, Vickie H. Y. Wong, Brad Fortune

**Affiliations:** ^1^Department of Optometry and Vision Sciences, University of Melbourne, Parkville, VIC 3010, Australia; ^2^Devers Eye Institute and Legacy Research Institute, Legacy Health, Portland, OR 97232, USA

## Abstract

Intraocular pressure (IOP) elevation is a key risk factor for glaucoma. Our understanding of the effect that IOP elevation has on the eye has been greatly enhanced by the application of the electroretinogram (ERG). In this paper, we describe how the ERG in the rodent eye is affected by changes in IOP magnitude, duration, and number of spikes. We consider how the variables of blood pressure and age can modify the effect of IOP elevation on the ERG. Finally, we contrast the effects that acute and chronic IOP elevation can have on the rodent ERG.

## 1. Introduction

The retina is unique in its specialisation for the capture of light and transduction into an electrical signal. Effective phototransduction and signal transmission involves many processes that are energetically expensive, including the maintenance of transmembrane ionic gradients, signal amplification, and neurotransmission. Not surprisingly, the retina is the most metabolically demanding of all the body's tissues [1, 2]. Given such a metabolic burden the neural retina may be uniquely susceptible to metabolic stress and disease. 

Glaucoma is the second leading cause of blindness in Australia [3] and worldwide [4], with intraocular pressure (IOP) being a key risk factor [5]. IOP reduction is an effective treatment regardless of the type of glaucoma [6, 7]. However, glaucomatous neuropathy can occur in the presence or absence of a detectable IOP elevation. The role that IOP-related stress may have in the development and progression of glaucoma is not fully understood. 

The retinal response to a flash of light is termed the electroretinogram (or ERG) and is a commonly used assay of retinal function. The ERG sums the field potentials of various retinal cells following light stimulation [8]. The ERG is noninvasive, easy to measure, and highly reproducible [9, 10] and is thus ideally suited to assess the retina as a whole. Decomposition of the overall voltage envelope into its constituent parts allows the underlying cell classes to be considered with some level of specificity [8]. a-wave and b-wave features of the bright flash ERG are excellent indicators of photoreceptoral and bipolar cell activity [11]. It needs to be mentioned that although the scotopic rat a-wave primarily reflects photoreceptor activation it also has inputs from second-order neurons that modulate the waveform beyond 14 ms [12]. Responses collected for very dim stimulus flash energies—equate to behavioral light detection thresholds [13]—are known as the scotopic threshold response (STR), which largely reflects the integrity of retinal ganglion cells in rodents [14, 15]. The high-frequency wavelets residing on the leading edge of the b-wave (oscillatory potentials, OPs) are a useful indicator of inner retinal activity including that of amacrine cells [16]. Thus in a relatively short recording period, information regarding the health of broad retinal cell classes can be collected. 

A more extensive discussion of the application of the ERG on rodents can be found elsewhere [17]. This paper describes the application of the ERG to study the effects of metabolic inhibition and IOP-related stress on the retina. The paper focuses on the effect of stress on the rat retina, an increasingly utilised model of retinal diseases, including glaucoma. 

## 2. Metabolic Stress and the ERG

Previous studies have shown that the ERG is exquisitely sensitive to metabolic stress. In general such studies show that the ERG is rapidly lost when normal metabolic substrate supply is interrupted, as occurs following hypoglycaemia [18], hypoxia [19, 20], and acute ischemia [21] most commonly achieved via interruption of the blood supply [22, 23]. 

**Figure 1:**
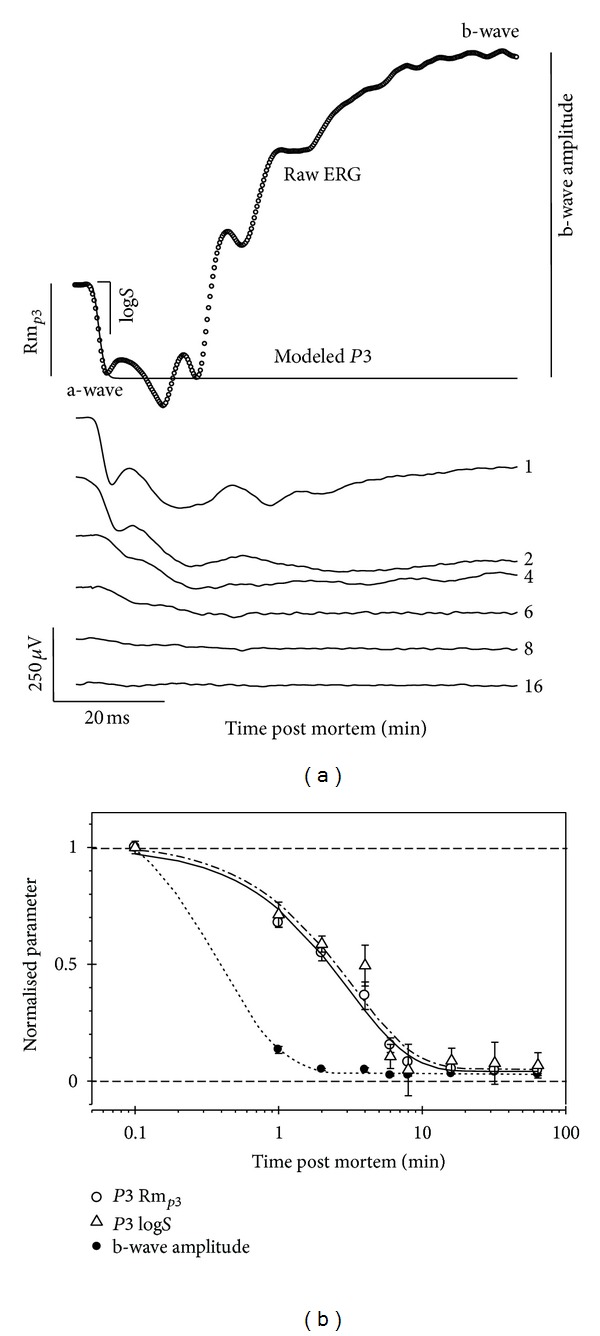
Retinal function is rapidly lost in the rat retina following acute complete inhibition of metabolite supply. (a) The leading edge of the raw response (a-wave, unfilled circles) was modeled using a phototransduction model (*P*3, thick line). (b) Postreceptoral b-wave (filled circles) is more susceptible than phototransduction amplitude (Rm_*P*3_, unfilled circles) and sensitivity (log⁡⁡*S*, unfilled triangles). Reprinted from [24] with permission. The Association for Research in Vision and Ophthalmology remains the copyright holder.

The rate at which retinal function declines after complete impairment of substrate supply can be interpreted as a measure of endogenous substrate availability (e.g., remaining adenosine triphosphate, oxygen, glucose, and glycogen).  Figure 1 shows that the dark-adapted photoreceptoral a-wave in Sprague-Dawley rats continues to be measurable for up to 10 minutes following complete ischemia [24].

The photoresponse can be described using a mathematical model based on known biochemical cascade of phototransduction to provide a measure of the saturated dark current (Rm_*P*3_) and phototransduction sensitivity (log⁡⁡*S*) or amplification [25, 26].  Figure 1(b) shows that  Rm_*P*3_  and log⁡⁡*S* decay at a similar rate following hypoxemic insult, suggesting that phototransduction gain depends on the same energy producing mechanisms that maintain the Na^+^/K^+^ ATPases involved in restoring the dark current. It is evident from Figure 1 that the ERG b-wave is diminished within 1 to 2 minutes following the onset of complete ischemia. These data are consistent with previous findings that the inner retina is more sensitive to metabolic stress than is the outer retina. 

The rate of ERG decline following metabolic challenge is constrained by the availability of remnant metabolic substrates including those that can be mobilised from endogenous stores. This explains the greater susceptibility of the b-wave to ischemic insult compared to the a-wave, as the photoreceptor sequesters its neurotransmitter (glutamate) to sustain metabolism at the expense of synaptic transmission [24]. Thus conditions that reduce the use of such substrates should delay the loss of function as previously suggested [27, 28]. One manner in which metabolic consumption can be reduced is via light adaptation, which has been shown to attenuate oxygen [29] and glucose consumption [30] particularly at photoreceptor inner segments [31]. 

**Figure 2:**
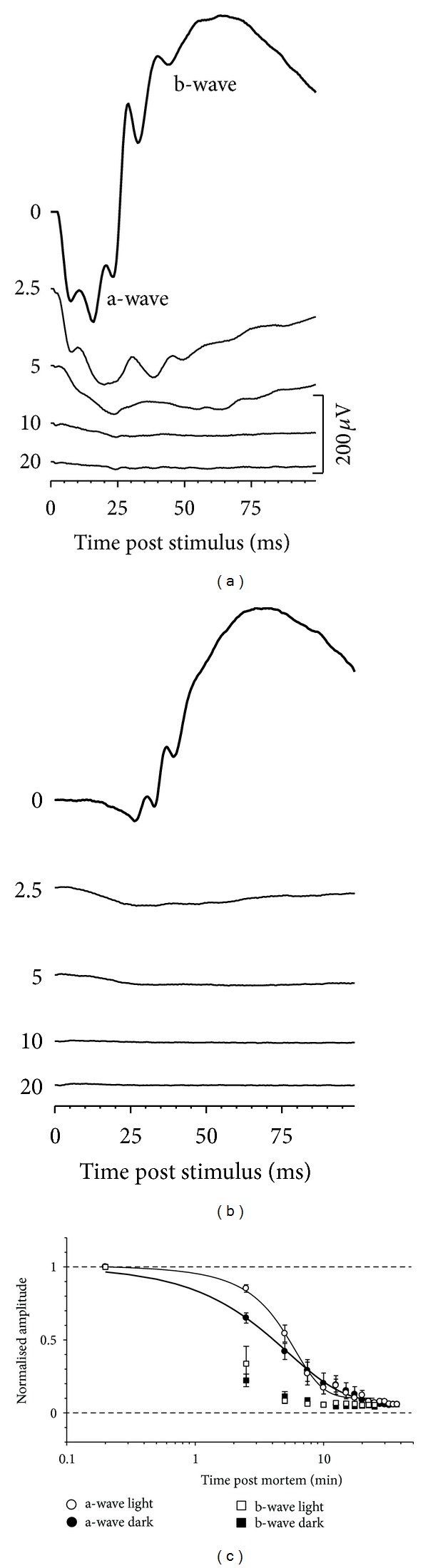
Light adapting the retina results in relatively slower decay of a-wave amplitudes at 2.5 and 5 minutes following ischemic insult. ERG loss under dark- (a) and light-adapted conditions (b). Group data are shown in (c). Reprinted from [24] with permission. The Association for Research in Vision and Ophthalmology remains the copyright holder.

Figure 2 shows that light-adapted ERGs in rats have a smaller a-wave and slower b-wave compared with humans [32] and nonhuman primates [33]. A smaller a-wave is consistent with fewer cones in rat retinae [34]. Similar to scotopic [35], and photopic [33] a-wave from nonhuman primates, the scotopic a-wave in rats [12] also contains contributions from hyperpolarising bipolar cells. Despite a smaller a-wave, the light-adapted b-wave in rats is almost 60% the size of its dark-adapted counterpart, consistent with the relatively large number of cone bipolar cells in the rat retina [34]. In comparison to the dark-adapted condition, the light-adapted a-wave was relatively less attenuated at 2.5 and 5 minutes after ischemic injury (Figures 2(b) and 2(c)). 

Our findings are consistent with previous reports that rod responses show greater susceptibility to hypoglycemia than light-adapted cone responses [36, 37]. Whilst both rods and cone may benefit from reduced metabolic demand associated with light adaptation, cones may inherently be less susceptible. For example, cones store glycogen, whereas rods lack glycogen and the rate-limiting glycogenolytic enzyme, glycogen phosphorylase [38, 39]. 

## 3. IOP Elevation and the ERG

Metabolic inhibition can also be induced by elevation of intraocular pressure above the mean arterial blood pressure [20]. Whilst this approach shares similarities with occlusive models of ischemia, IOP elevation also increases mechanical load on retinal tissues [22, 23].

The balance between the production and outflow of aqueous humor determines intraocular pressure. This nutritive fluid supports the metabolism of most avascular tissues of the eye. It is actively secreted by the epithelium of the ciliary body into the posterior chamber. From the posterior chamber aqueous moves down an osmotic gradient through the pupil to enter the anterior chamber. The primary outflow pathway is through the trabecular meshwork, with a small percentage (10%) exiting *via* the uveoscleral route. Thus, any resistance in the outflow (e.g., blockage in trabecular meshwork) or a change in the anatomic relationship between the pupillary margin and the lens will result in IOP elevation [40–42]. 

Whilst it is clear that elevated IOP is an important risk factor for glaucoma [43], the elevated IOP hypothesis fails to account for the fact that many glaucoma patients (20–30%) have normal pressure [5, 44]. In addition, the disease can progress in 20% of eyes even though IOP has been substantially (≥30%) lowered [45], whereas over half of untreated individuals with ocular hypertension do not show visual field progression [44, 45]. Such apparent clinical paradoxes may arise from an incomplete understanding of how change in IOP affects visual function and neuronal integrity. Thus a deeper understanding of how IOP influences the integrity of the retina is crucial to understanding the pathophysiology of glaucoma. We have considered these issues with an acute IOP insult as will be described next.

**Figure 3:**
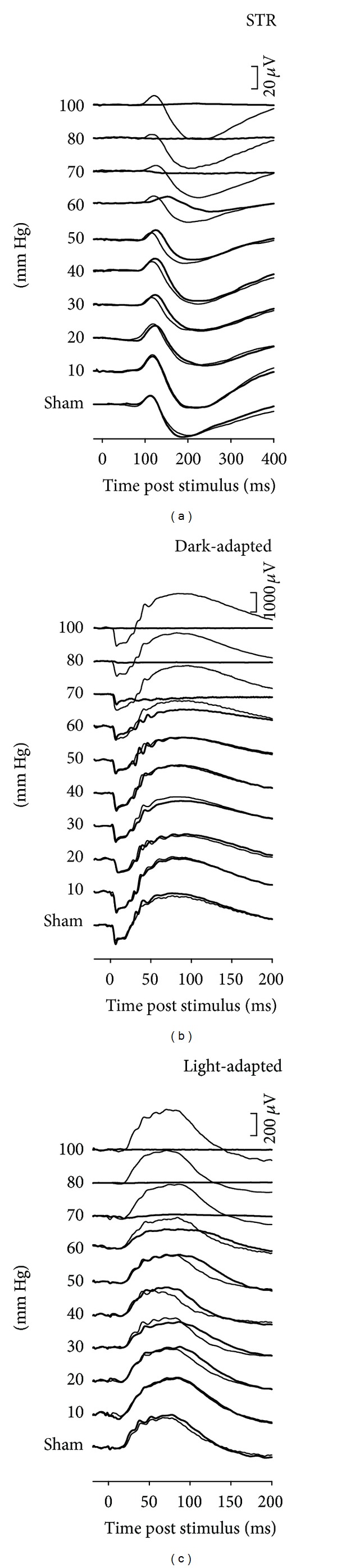
Rat ERG response to various levels of acute IOP elevation. (a) Scotopic threshold response (STR, −5.55 log cd s m^−2^). (b) Scotopic bright flash ERG (2.22 log cd s m^−2^). (c) Photopic ERG (2.72 log cd s m^−2^ on a steady white background of 150 cd·m^−2^). ERG responses for fellow control eyes are shown by the thin traces in each column. Reprinted from [46] with permission. The Association for Research in Vision and Ophthalmology remains the copyright holder.

Figure 3 shows that acute IOP elevation produces a dose-related decline in the ERG. During IOP elevation reductions in ERG amplitude are not seen until IOP is elevated to 60 mmHg or more. For pressures beyond 60 mmHg the ERG shows progressively greater attenuation and is abolished at 100 mmHg. 

### 3.1. Which Component of the ERG Is Most Sensitive to IOP Elevation?

A number of studies have compared the effect of IOP elevation on ERG components whose cellular origins have been attributed to outer, middle, and inner retina. These studies are in agreement that the outer retina is less sensitive to IOP elevation than is the inner retina. Foulds and Johnson [47] reported that the a-wave recovered faster than the b-wave following IOP elevation. Furthermore, comparison between outer retinal and ganglion cell function achieved by simultaneously measuring the flash ERG (b-wave) and pattern ERG, respectively, are consistent with greater inner retinal sensitivity to IOP elevation. Specifically, both chronic IOP elevation in monkeys [48] and acute IOP elevation in cats [49] and rabbits [50] resulted in decreased pattern ERG amplitudes, whereas the ERG b-wave remained unchanged.

**Figure 4:**
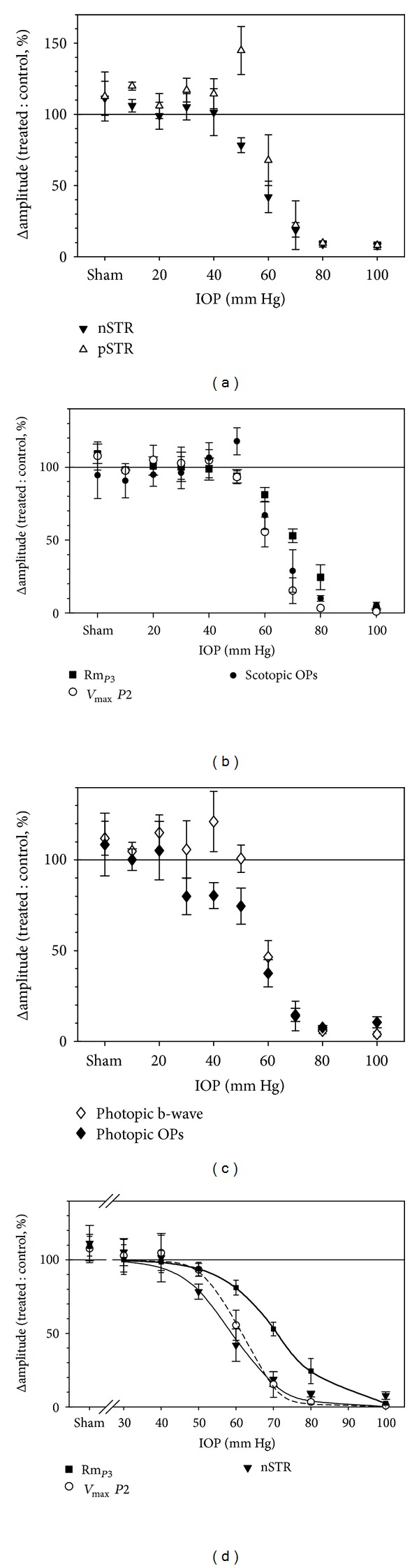
Relative change in ERG amplitude versus IOP (mean ± SEM). (a) Positive STR (pSTR, unfilled triangles) and negative STR (nSTR, filled triangles). (b) Photoreceptoral amplitude (Rm_*P*3_, filled squares),  *P*2  amplitude (*V*_max⁡_, open circles), and scotopic OP amplitude (filled circles). (c) Photopic b-wave amplitude (open diamonds) and photopic OP amplitude (filled diamonds). (d) nSTR (filled triangles, thin solid curve),  *P*2  *V*_max⁡_ (open circles, dashed curve), and photoreceptoral  Rm_*P*3_  (filled squares, bold curve) are replotted together with their corresponding best-fit cumulative normal functions for comparison. Reprinted from [46] with permission. The Association for Research in Vision and Ophthalmology remains the copyright holder.

As shown in Figure 3 and summarised in Figure 4 the nSTR and photopic OPs of full-field ERG are most sensitive to acutely elevated IOP. The photopic b-wave was the third most sensitive component by amplitude analysis, as it was reduced by 50% for an IOP level of ~60 mmHg. We have previously shown that the STR, photopic b-wave, and photopic OPs were the most sensitive components of the rat full-field ERG to removal of intact ganglion cell function, via optic nerve transection and intravitreal injection of tetrodotoxin [14]. The finding that acute IOP elevation has the largest impact on all of these full-field ERG components suggests that the elements most sensitive to acutely elevated IOP in the rat ERG arise from the inner retina, and in particular intact ganglion cell function (also see Figure 6). This pattern of deficit is consistent with the idea that the large volume and high flow of the choriocapillaris provide a robust buffer against IOP elevation in the rat thereby preserving outer retinal components. The resistance of the rat choroidal perfusion to IOP elevation has been recently demonstrated using optical coherence tomography/optical microangiography [51].

### 3.2. How Quickly Does the ERG Recover from Stress? 

Central to the capacity of the eye to resist injury is its ability to recover from stress. In response to complete ischemia induced by IOP elevation (100 mmHg for 60 minutes) our group, as well as others, have shown that the ERG does not recover fully. Indeed, there is often a severe reduction of the ERG b-wave a week after the insult [22, 23, 52]. As was the case during IOP elevation ERG components arising from the outer retina show better recovery than do those generated by the inner retina. It is of interest that ischemia-reperfusion produces a relatively greater effect on the cone ERG [53] isolated using a paired or twin flash paradigm [54–56]. Why reperfusion injury should affect the cone ERG more than the rod ERG, when the converse was the case during ischemic stress (Figure 2) is not clear. 

**Figure 5:**
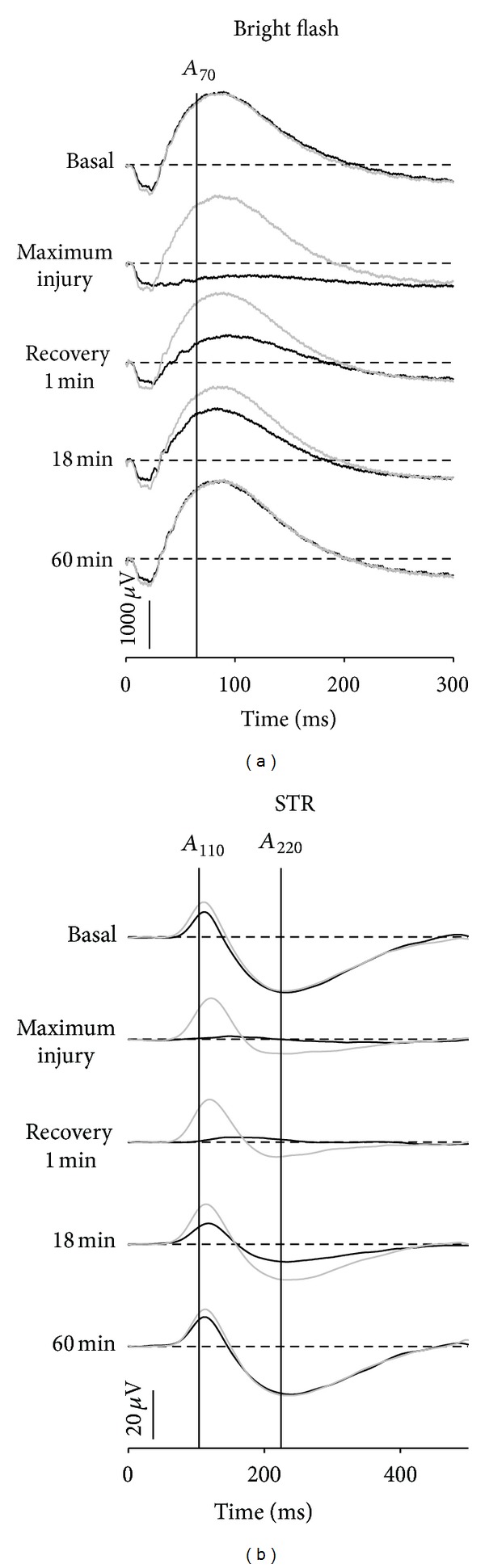
Recovery of bright flash ((a)  1.0log⁡ cd · s · m^−2^) and STR waveforms ((b)  −4.8log⁡ cd · s · m^−2^) from IOP elevation to 50 mmHg for 42 min (greyed traces) and 70 mmHg for 30 min (thick traces). Reprinted from [57] with permission. The Association for Research in Vision and Ophthalmology remains the copyright holder.

In contrast, lower levels of IOP elevation produce reversible ERG losses, as illustrated in Figure 5. Indeed, once IOP had been returned back to baseline the ERG rapidly recovers. Overall there was more severe dysfunction during IOP elevation at 70 mmHg compared with 50 mmHg. For IOP elevation to 50 mmHg (42 minutes) the ON-bipolar cell  *P*2  had largely recovered within a minute after restoration of normal IOP. The inner retinal nSTR took more than 45 minutes to show a similar relative level of recovery. Both bright flash (a-wave and b-wave, Figure 5(a)) and STR responses (Figure 5(b)) take longer to recover in the case of more severe ERG attenuation (70 mmHg). 

**Figure 6:**
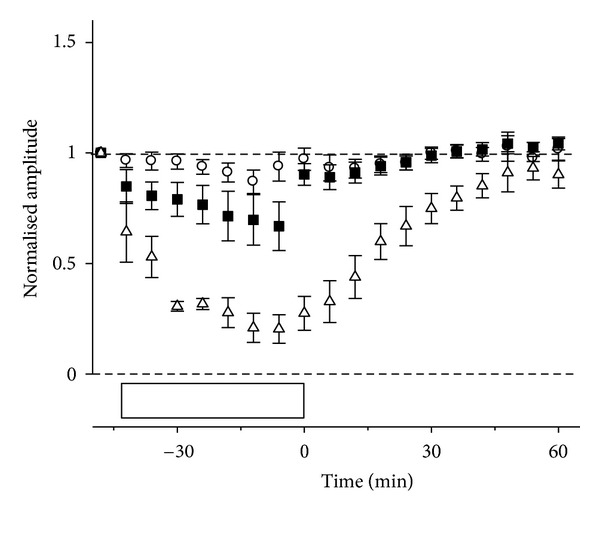
Relative amplitude of ERG components (mean ± SEM) during and after IOP elevation to 50 mmHg for 42 min.  *P*3  (circles),  *P*2  (squares), and nSTR (triangles) amplitudes are compared. Horizontal reference lines indicate baseline and zero levels. Open boxes show the duration of IOP elevation. Reprinted from [57] with permission. The Association for Research in Vision and Ophthalmology remains the copyright holder.

The above data show that the severity of injury, as indicated by the magnitude of ERG amplitude attenuation, determines how long it takes to recover from stress. Figure 6 confirms that the component of the ERG that recovers fastest is that which was least affected, the photoreceptoral  *P*3. The ON-bipolar cell  *P*2  is the next to recover and finally the inner retinal nSTR. Thus the sensitivity of the inner retina to IOP elevation is expressed in two ways, first a larger magnitude of ERG reduction and second a delay in recovery. Both of these are useful indices of the retinal capacity to overcome stress.

Several factors may account for the greater sensitivity of the inner retina to IOP elevation. IOP elevation has the potential to produce both vascular and mechanical stress at the level of the retina and particularly the optic nerve head. The presence of optic nerve stress would have a greater impact on ganglion cell function compared with ERG components arising from middle and outer retinal layers. Moreover, a number of studies have shown that IOP-related stress (force/cross-sectional area) is magnified at the optic nerve head in nonhuman primates [58, 59] and rats [51, 60]. An altered pressure gradient across this tissue makes ganglion cell axons and small blood vessels traversing the optic nerve head particularly susceptible to IOP-related stress.

### 3.3. Which Is More Detrimental to the ERG, the Magnitude or the Duration of IOP Elevation? 

**Figure 7:**
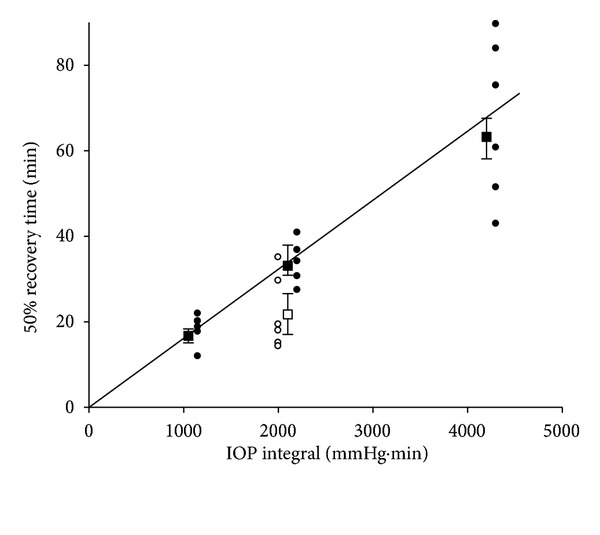
Relationship between the 50% recovery time (mins) for nSTR amplitude as a function of IOP time integral for elevation to 70 (filled symbol) and 50 mmHg (unfilled symbols). Individual animals are shown, and squares indicate bootstrapped mean for group data; error bars, 2.5% and 97.5% bootstrap confidence limit. Reprinted from [57] with permission. The Association for Research in Vision and Ophthalmology remains the copyright holder.

Whilst it is clear that the magnitude of IOP elevation has a profound effect on the ERG, there is growing recognition that the duration of IOP elevation is also important. The concept of the total integral of injury (pressure × duration) accounts for substantial variation in the degree of functional and histological damage in various models of chronic IOP elevation [61–65]. We have shown that the grade of optic nerve injury was more closely related to the mean IOP than the peak IOP in rats with chronic IOP elevation [66]. Another way to try and disentangle the effects of IOP magnitude from duration is to consider the effect of IOP elevations of different magnitude but of equivalent IOP integral.  Figure 7 plots the time taken for 50% ERG recovery as a function of IOP integral (mmHg·min). The filled symbols show that at an IOP of 70 mmHg the time taken for recovery is progressively prolonged as the integral increases. Also plotted on the figure is an IOP elevation to 50 mmHg for a longer time (42 minutes) but which produces the same integral as a 70 mmHg IOP spike of 30 minutes duration (2100 mmHg·min). It is clear that for IOP spikes having equivalent integrals, (as given by IOP magnitude ∗ time) the higher IOP magnitude produces a greater delay in the rate of recovery. 

### 3.4. Are Repeated IOP Elevations Worse Than a Single Event?

Drance [67] proposed that undetected IOP fluctuations occurring outside of office hours may be responsible for, or contribute to, glaucoma progression. It is well known that IOP has a circadian rhythm in both normal subjects and glaucoma patients [68, 69]. Peak IOP often occurs during the night or towards the early morning [69, 70] in various species, including humans [69], rabbits [71], rats [72], and mice [73]. Saccà et al. [74] report that IOP fluctuations are greater in glaucoma patients compared with age matched controls. A number of studies have suggested that greater IOP fluctuation increases the risk of visual field progression [75, 76]. Others have failed to find such an association [77].

**Figure 8:**
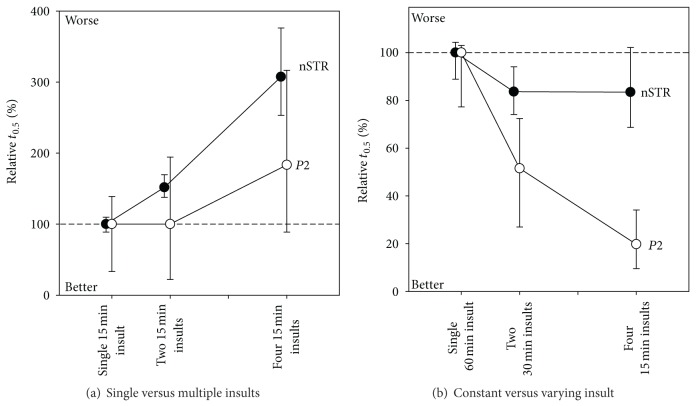
Effect of repeated IOP elevation on ERG recovery. The time taken for 50% (*t*_0.5_) recovery following repeated IOP insults is expressed relative to a single IOP insult (±95% CI) for ON-bipolar cell b-wave (*P*2, unfilled symbols) and inner retinal responses (nSTR, filled symbols). (a) Comparison of single and multiple insults at the same IOP. (b) Comparison of single and multiple insults of equal integral. Reprinted from [78] with permission. The Association for Research in Vision and Ophthalmology remains the copyright holder.

We considered this issue by evaluating recovery of the ERG following IOP insults of fixed magnitude and duration but varying the number of insults.  Figure 8 shows the time taken for 50% amplitude recovery expressed as a percentage relative to a single IOP insult.  Figure 8(a) shows that recovery of the rat ERG after multiple IOP insults becomes slower as the number of insults increases. The exacerbation of functional deficit is more pronounced for the nSTR compared with the  *P*2, again highlighting the sensitivity of the inner retina. 

That recovery is progressively prolonged as the number of insults increased is expected as the IOP integral also increases, and we have shown in Figure 7 that IOP integral has a linear relationship with recovery time. A more appropriate way to consider the importance of repeated IOP insults is to match the insults for their total duration (IOP ∗ time integral). That is, will a single spike of 60 minutes produce the same insult as 4 spikes each of 15 minutes? Figure 8(b) shows that a prolonged single 60-minute insult produces more delay in ERG recovery when compared with two 30-minute and four 15-minute insults. Figure 8(b) also shows that the benefits are most marked for the bipolar cell-derived  *P*2  and not the ganglion cell-derived STR.  Figure 8 provides evidence that number of insults is particularly detrimental to retinal ganglion cell function, which does not benefit from shorter durations in IOP elevation. 

This leads to the interesting observation that the time taken for nSTR recovery was similar, regardless of the number of insults, as long as the integral was kept constant. In contrast the bipolar cell  *P*2  recovered faster when the insult was divided into multiple short-duration episodes. Taken together, the data indicates that compared to the middle retina, the inner retina does not cope as well with repeated IOP insults.

### 3.5. What Underlies the Loss of the ERG Occurring during IOP Elevation?

**Figure 9:**
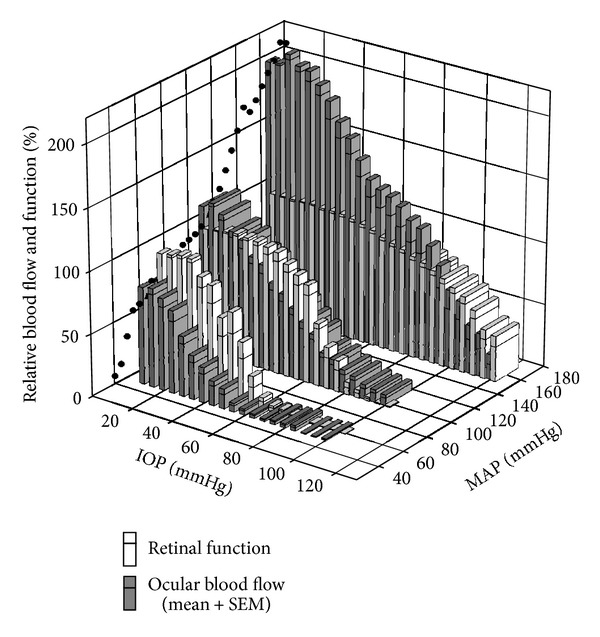
Comparison of relative retinal function and ocular blood flow during IOP elevation in animals with acute low, moderate, and high blood pressure. Black circles: blood flow autoregulation curve at baseline IOP. Function (unfilled bars, b-wave amplitude) is normalized to baseline. Starting blood flow (filled bars) is adjusted based on the autoregulation curve. Bars represent mean + SEM. Reprinted from [89] with permission.

Elevated IOP is thought to cause retinal damage by mechanical and vascular mechanisms. Vascular injury is likely to arise from direct compression of blood vessels in the optic nerve head and retina. When IOP elevation reduces ocular perfusion pressure (OPP) beyond the capacity for autoregulation, ocular blood flow will be reduced thus producing retinal dysfunction. Consistent with this idea, Figure 9 shows that the ERG amplitude and ocular blood flow decrease as IOP increases. 

More interestingly these data show that, for any given IOP, blood flow was relatively more affected than retinal function. These data suggest that some other factor helps to sustain function despite relative ischemia. Thus the ERG does not require 100% blood flow to remain within normal limits. This outcome suggests that there is range of blood flow that is adequate to supply the energetic needs of the ERG. Previous studies have shown that oxygen rather than blood flow is critical for the health of the retina [20, 79–81] and optic nerve [82]. One mechanism that may help to maintain tissue oxygenation in the presence of IOP-induced reductions in blood flow is to increase oxygen extraction from residual arterial blood during mild ischemia. Data from human cerebral [83–85] and muscular [86, 87] circulation consistently show that oxygen extraction increases with decreased blood flow. Tornquist and Alm [88] have shown in porcine retina an increased difference in arterial venous oxygen tension during reduced ocular perfusion, consistent with increased oxygen extraction under these conditions. Whether changes in oxygen extraction occur in chronic disease such as glaucoma requires further study. 

## 4. Other Factors Modifying the Response of the Rat ERG to IOP Elevation 

### 4.1. How Does Blood Pressure Influence the ERGs Response to IOP Elevation?

As OPP expresses the balance between mean arterial pressure and IOP (OPP = MAP − IOP), a reduction in systemic blood pressure (BP) or a comparable increase in IOP should have similar effects on retinal function. For this reason there is increasing awareness that BP and thus OPP are important in glaucoma [90, 91]. Consistent with the above idea low BP has consistently been found to be a risk factor in glaucoma [92–99] and has shown to undermine normal blood-flow autoregulation within the primate optic nerve head [100]. At the other extreme, high BP has been reported to lower the risk of glaucoma [97, 99, 101, 102]. However, a host of other studies [98, 103–106] report that hypertension increases glaucoma risk. 

Given the above, a better understanding of how IOP and BP interact to influence retinal function is needed. An acute experimental approach that affords accurate simultaneous control of both IOP and BP is useful as it avoids long-term cardiovascular confounds. This was recently achieved by measuring retinal function and blood flow over a wide range of OPPs, induced by IOP elevation in rats with low, moderate and high levels of acutely modified BP (Figure 9). In particular, pharmacological manipulation was employed to clamp BP at low and high BP levels for the duration of the IOP challenge. Figure 9 shows that in comparison to rats with normal BP, those with low and high BP show increased and decreased susceptibility to IOP elevation, respectively. This was true for both the b-wave amplitude and ocular blood flow. However, when function and blood flow are plotted as a function of OPP rather than IOP for the three BP groups, they do not overlie each other. Indeed, higher IOPs produced greater dysfunction even though the OPP was the same. It is likely that the additional mechanical load on the eye produced by the IOP elevation produces departures from the predicted OPP relationship due to mechanical effects. That is OPP should only be considered as a measure of vascular stress and may not be the metric that best describes glaucoma risk. The increased mechanical load leading to changes in the shape of the eye and optic nerve head may confer additional acute and chronic stresses on retinal ganglion cell axons and glia within the optic nerve head and in other peripapillary tissues. 

### 4.2. Do Other Risk Factors Modify the ERGs Response to IOP Elevation?

The above studies show that the ERG, in particular those components that arise from progressively more proximal retinal generators, are excellent measures of retinal stress when used in combination with a short IOP spike. This type of “stress test” is in some ways analogous to that used to assess the risk of cardiovascular disease. This concept has been applied in the clinic in an attempt to develop a more sensitive test of glaucoma. For example studies have measured the ERG during temporary IOP elevation using suction cup [107–110] or postural inversion [109, 111–113]. 

We adopt a similar approach in rodent models in order to assess how additional risk factors might influence the eye's response to IOP elevation. As discussed previously we find that the most sensitive metric of stress is the extent to which functional recovery is delayed and not the amount of functional loss. Indeed, repeated sampling during a protracted recovery allows the process to be quantified, thus reducing the variability associated with a single ERG measurement. Moreover, this approach using mild insult allows subtle differences in susceptibility to be revealed in a relatively rapid experimental paradigm [114]. As described below, we apply this approach to study the effect of aging on IOP susceptibility. 

 Advancing age is a major risk factor for glaucoma [115]. An emerging theory is that age-related mitochondrial dysfunction increases the vulnerability of neurons to injury [116]. We show that compared with 3-month-old C57 mice, older animals suffered greater decline in retinal function and slower recovery following IOP of 50 mmHg for 30 minutes. This increased susceptibility is evident in 18-month-old mice. In the same study we were also able to show that alternate day fasting could ameliorate the increased susceptibility to the IOP stressor. This improvement in function was associated with increased mitochondrial enzyme expression and activity [117]. 

We undertook a parallel study in a murine model of accelerated aging. Polymerase  *γ*  (PolG) [118, 119] is an enzyme that proofreads mitochondrial DNA as it is replicated [120]. Mice carrying systemic mutations in the proofreading domain of PolG [121, 122] exhibit premature senescence. We show that by 12 months of age PolG mice are more sensitive to IOP elevation [123]. These data provide support for the idea that mitochondrial dysfunction increases the susceptibility of the retina to IOP elevation. 

## 5. The ERG in Chronic IOP Elevation

The response to a single mild IOP insult at best models the very earliest and reversible stages of injury that may ultimately contribute to ganglion cell loss in glaucoma. Not surprisingly chronic mild IOP elevation over the course of weeks produces a distinct pattern of ERG changes. 

We have shown that chronic IOP elevation, induced by hypertonic saline injection into the episcleral veins in rats, produces a wide severity of retinal dysfunction [66]. The level of severity was best correlated with the mean IOP measured noninvasively four to five times a week over the six weeks of chronic IOP elevation. ERG deficits also showed a modest correlation with the severity of optic nerve damage assessed using gross histology. 

**Figure 10:**
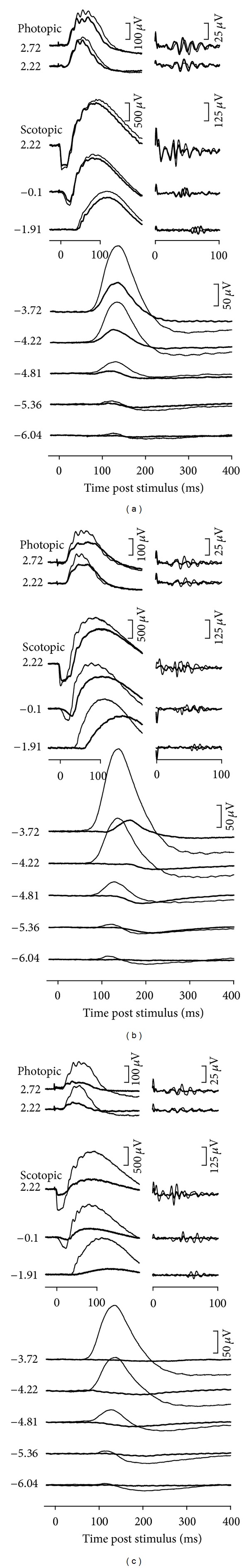
Effect of chronic IOP elevation on the rat ERG. Representative individual examples of ERG findings for experimental eyes (bold traces) and fellow control eyes (thin traces). Examples are given for eye showing mild (a), moderate (b), and high (c) IOP elevation. Stimulus flash intensities are listed at left for scotopic and photopic responses. Isolated OPs are shown to the right of corresponding waveforms. Reproduced with permission from [66].

Importantly we were able to show from data similar to that in Figure 10 that chronic IOP eyes could be grouped into those showing ERG dysfunction in the presence and absence of gross morphological change. These ERG deficits appeared to be greater for the pSTR (with some effect on the nSTR and the photopic b-wave) with little change to the a-wave or b-wave. In general, these animals had a mean IOP less than 30.2 mmHg. Eyes with clear signs of optic nerve changes tended to have higher mean IOP (mean 31.1 mmHg) and showed more severe ERG deficits, including almost complete loss of the pSTR, a marked reduction in nSTR, b-wave and scotopic and photopic OPs, with only a small effect on the ERG a-wave. Finally, in those animals showing higher IOP (mean 37.8 mmHg) and severe widespread optic nerve changes, the pSTR was completely abolished, and a- and b-waves, as well as photopic b-waves, were all reduced to less than 30% of control eye values.

### 5.1. How Do ERG Changes Differ in Acute and Chronic IOP Elevation?

The above outcome suggests that selective RGC damage, as evident by attenuation of the STR, is produced by a relatively mild chronic elevation of IOP, whose mean over the six weeks of the experiment was less than 30 mmHg. This is consistent with the data for acute IOP elevation, where functional changes could be observed at an IOP of 30 mmHg. In particular, at 30 mmHg a delay in the pSTR as well as in the trailing edge of the photopic ERG was evident, with little change to the bright flash a-wave or b-wave. Amplitude losses (nSTR) do not become apparent until IOP is elevated at least to 50 mmHg (Figure 4). 

The ERG deficits even at this low level of IOP elevation are associated with a mild decline in ocular blood flow, as shown in Figure 9 and more recently by Zhi et al. [51]. Thus it is likely that both mechanical and vascular stress is responsible for the dysfunction observed in both acute and chronic IOP elevation. What is likely is that the relative proportion of injury attributable to these mechanisms will differ in the two cases. Clearly dysfunction associated with acute IOP elevation lacks potential sources of stress arising from tissue remodelling of the optic nerve head associated with chronic IOP elevation. 

A single IOP spike of 30 mmHg is clearly not enough to cause permanent dysfunction as we have shown that the ERG completely recovers from short IOP spikes of 50 mmHg and even 70 mmHg (Figure 5). It is likely that chronic IOP elevation even at this mild level does not give the retina time to recover, thus leading to cumulative dysfunction.  Figure 8(a) shows that repeated insults will progressively lengthen the time taken for recovery, particularly for the STR. One might extrapolate this to an extreme scenario whereby no recovery is possible.  Figure 8(a) also demonstrates the idea that the accumulation of dysfunction will be more profound for the STR compared with the b-wave. 

In general acute (Figure 4) and chronic IOP effects produce a similar pattern of loss to ERG components. That is, those components arising from the inner retina are more attenuated. An interesting difference between acute and chronic IOP effects is that the nSTR and the pSTR show greater sensitivity to the two conditions, respectively. In the acute case (Figure 4) the most sensitive component of the ERG is the negative component of the STR, whereas in the chronic situation the pSTR shows the largest deficit (Figure 10). The reasons for this are not clear. Our study employing optic nerve transection and tetrodotoxin application in rats suggests that the pSTR is almost entirely ganglion cell dependent in this species. On the other hand, the nSTR was not completely abolished following the same treatments suggesting that it contains “non-ganglion cell” or tetrodotoxin-insensitive [14] contributions. One might speculate that such elements are sensitive to acute IOP elevation thus contributing to the greater attenuation of the nSTR during acute stress. Potential candidates include amacrine [124] and glial cells [125] both of which are known to be sensitive to acute hypoxic and hypoglycemic stress. 

## 6. Future Directions

The above discussion shows that the magnitude, the duration, the frequency, and the recovery time between subsequent stressors and chronicity of IOP elevation all influence the functional integrity of the retina. The studies described above are by no means comprehensive and sample only a limited set of variables in a complex biological system. What remains to be defined is the exact IOP integral above which the retina becomes irreversibly damaged. Indeed, the idea of IOP integral as it relates to the point of irreversible damage needs to be revisited. Given our findings, it seems that IOP magnitude needs to be weighted more than the duration of stress. Moreover, it is likely that the weighting would need to be modified if IOP elevation were to occur in the setting of eyes with impaired autoregulation (e.g., diabetes, systemic hypertension, and vasospastic disease) or those prone to manifest more stress and strain at the optic nerve (e.g., altered biomechanics of optic nerve connective tissue in ageing, in myopia).

Clearly ERG dysfunction is related to some form of vascular stress. However, an explanation based purely on the vascular supply as expressed by OPP = BP − IOP is inadequate to account for the spectrum of functional changes at various BPs. Indeed, a simple change in blood flow fails to explain ERG loss, as function seems to be robustly buffered against mild ischemia. There are clearly other regulatory mechanisms such as those involving oxygen extraction and local metabolic stores that need to be considered. What is needed is a study that simultaneously quantifies the effect of IOP elevation on the ERG, blood flow, and retinal oxygen tension. Such an approach would provide insights into the mechanisms that protect the retina from irreversible injury.

Whilst it is clear that acute changes in BP substantial modify the susceptibility of the ERG to IOP elevation (Figure 9), it is not known what effect chronic hypertension would have. The data of Figure 9, which are derived from acute manipulations, suggest that hypertension should be protective against elevated IOP. However, a number of epidemiological studies report that long-standing systemic hypertension increases the risk of glaucoma [98, 103–106]. Thus it will be important to assess the effect of IOP elevation in a model of chronic systemic hypertension. Moreover, the effect of chronic hypertension needs to be examined in a chronic model of glaucoma in order to provide greater clinical relevance.

## 7. Summary

In summary the ERG provides an excellent index of retinal stress. It is exquisitely sensitive to metabolic stress and IOP elevation and is useful for both acute and chronic studies in rodents. Employing ERG components arising from the inner retina along with a mild IOP elevation allows risk factors to be assessed in a controlled manner in rodent models of glaucoma. The development of a noninvasive means to elevate IOP in the rodent eye would allow for the application of a “stress test” to be employed in longitudinal assessment of preclinical disease models and give an indication of the effectiveness of their treatments.
